# A pilot study: relationship between Bisphenol A, Bisphenol-glucuronide and total 25 hydroxy vitamin D in maternal-child pairs in a South African population

**DOI:** 10.3389/fendo.2024.1108969

**Published:** 2024-11-28

**Authors:** Verena Gounden, Rajen N. Naidoo, Anil Chuturgoon

**Affiliations:** ^1^ Department of Chemical Pathology, University of KwaZulu-Natal and National Health Laboratory Services, Inkosi Albert Luthuli Central Hospital, Durban, South Africa; ^2^ Discipline of Occupational and Environmental Health, University of KwaZulu-Natal, Durban, South Africa; ^3^ Department of Medical Biochemistry, University of KwaZulu-Natal, Durban, South Africa

**Keywords:** Bisphenol-A, vitamin D, high performance liquid chromatography, mother-child pairs, cord blood

## Abstract

**Introduction:**

Exposure to Bisphenol A (BPA) during early development particularly *in-utero* has been linked to a wide range of pathology. Over the last two decades the importance of vitamin D in maternal and child health has been highlighted. The aim of this pilot study was to examine the relationship of BPA and its naturally occurring metabolite BPA-glucuronide (BPA-g) with 25-hydoxy vitamin D (25OHD) levels in South African mother-child pairs.

**Methods:**

Third-trimester serum maternal samples and matching cord blood samples were analyzed for BPA and BPA-g using liquid chromatography tandem mass spectrometry (LC-MS/MS) and 25OHD3 and 25OHD2 using high performance liquid chromatography. A total of 58 maternal and child pairs were analyzed.

**Results:**

More than fifty percent of maternal-child pairs were noted to be vitamin D deficient or insufficient using the Endocrine Society Practice guidelines cut-off of 50 nmol/L. Spearman rank correlation and Kruskal Wallis analysis did not show statistically significant relationship between cord 25OHD (total) and maternal and cord BPA-g concentrations. Analysis of covariance after controlling for confounders showed a significant relationship between cord BPA-g levels and cord 25OHD levels (p=0.03) as well as between maternal BPA-g levels (p=0.04) and cord total 25OHD levels (p=0.04).

**Discussion:**

The findings of the current study indicate a possible relationship with BPA/BPA-g and fetal/early infant Vitamin D levels that needs to be further investigated in this population.

## 1 Introduction

Vitamin D is a cholesterol derived prohormone that is available in two common storage forms, the plant derived, ergo-cholecalciferol (25 hydroxy vitamin D2) and the animal derived cholecalciferol (25 hydroxy vitamin D3) (1, 2). In humans, vitamin D3 is converted to the active form 1,25 hydroxy vitamin D which acts via vitamin D binding receptors in various tissues (2–5). Classically vitamin D has long been associated with bone and skeletal health, including calcium and phosphate metabolism (2). There are numerous studies that support the importance of vitamin D in other aspects of health including the development and maintenance of the immune system, neurodevelopment and development of reproductive organs. Inadequate levels of Vitamin D have been associated with tumorigenesis, abnormalities in glucose metabolism, cardiovascular disease, obesity and development of autoimmune disease (6, 7)

Vitamin D levels during the intrauterine and neonatal period are largely dependent on maternal vitamin D levels (8–10). Maternal vitamin D deficiency (VDD) can result in neonatal hypocalcemia. In addition, maternal VDD has been associated with other gestational pathologies including poor placentation and maintenance of the pregnancy, maternal obstetric complications such as preeclampsia, preterm birth as well as deleterious effect on fetal immune, neural and metabolic development. Maternal VDD may also negatively affect the anthropometric parameters in the neonate and increase the risk for asthma and type 1 diabetes in later life (11–14). Recent literature from different regions have reported significant proportions of neonates with insufficient 25 hydroxy-vitamin D (25OHD) levels and a similar prevalence in the mothers (15–22). However, the issue of VDD is clouded by lack of consensus for optimal cut offs to denote deficiency in young infancy and the possibility of different cut offs based on racial groups. Limited data is available on maternal and neonatal vitamin D status in the African continent and associated deleterious effects. A study performed in a Kenyan pregnant cohort reported a 51% Vitamin D insufficiency and a 21% deficiency (using Endocrine Society Guidelines) (23). However, a study in a Zimbabwean cohort did not show similarly high levels of deficiency/insufficiency (24).

The intra-uterine period is highly susceptible to the effect of endocrine disruptors on fetal health. Bisphenol A (BPA), an environmental chemical and endocrine disruptor, is found in a vast array of plastic consumer products including lining of tin cans, food and water containers, medical devices and toys (25, 26). Exposure to BPA has been linked to prenatal and postnatal adverse effects on multiple tissues, including the reproductive system and neurodevelopment. BPA effects are typically attributed to its estrogenic or anti-estrogenic action, however this action can not completely account for the adverse effects of low potency BPA at the low-dose exposures that are commonly seen (25, 27). BPA has also been reported to interact with other steroid hormone receptors including androgens and glucocorticoids (28). The structural homology of vitamin D with sex steroid hormones like estradiol and testosterone, make it possible that BPA may also disrupt the actions of Vitamin D as well.

A 2016 report from the US National Health and Nutrition Examination Survey (NHANES) examined the relationship between urinary BPA (uBPA) and 25OHD levels in a large cohort of pregnant women. Their findings showed an inverse association with uBPA and total 25OHD levels. BPA was significantly associated with a 20% increase in the odds of VDD at 26 weeks gestation in the women (29).

BPA-glucuronide (BPA-g) is a major metabolite of BPA metabolism. Levels in cord blood remain steady and are thought to reflect the cumulative does of BPA received during late pregnancy (30). BPA-g, was widely thought of inert however recent evidence in animal studies suggest that it may deconjugate to expose the fetus to BPA even though adequate conjugation of BPA has occurred after maternal intake (31). In context of the short half-life of BPA, BPA-g acts as a surrogate marker of BPA exposure (31).

In a previously published study, we reported BPA was detectable in more than 25% of maternal and cord blood samples in a South African cohort. We demonstrated significant positive correlation between maternal and child BPA and BPA-g levels with correlation coefficients of 0.892 and 0.744, respectively (32). As part of a larger study examining the effect of BPA on maternal and child-pairs we examined the relationship between BPA and BPA-g levels on 25-hydroxy Vitamin D levels in maternal and child pairs. As a secondary objective in this pilot study we also examined the relationship between 25 OH Vitamin D levels and birth parameters.

## 2 Methods

### 2.1 Population and study samples

A subset of blood samples and data collected as part of the Mother and Child in the environment (MACE) birth cohort study were utilized for this study. The MACE study population consists of “healthy” pregnant females recruited from antenatal clinics in industrially dense south Durban, South Africa and other clinics in the north Durban area. The south Durban is an area where large communities are located within heavily polluted large-scale industrial enterprises. The north communities, although of similar socio-economic profile, are less industrially active. Mothers were recruited during the third trimester of pregnancy. The overall objective of the MACE study was to describe birth outcomes among pregnant mothers in communities exposed to industrial pollution compared to communities without such exposure. Details regarding pregnancy outcome, smoking status and anthropometric measurements for participants were also collected during the course of the MACE study (33). Venous blood samples were taken during the third trimester (between 27 completed to 40 weeks of gestation) from the pregnant individuals. Cord bloods were taken at delivery. Bloods were collected from maternal participants at one of their regularly scheduled ante-natal appointments. Maternal and cord bloods collected in serum polypropylene vacutainer tubes were later analyzed for BPA and BPA-g. These samples were centrifuged, separated and serum stored at -80 degrees Celsius until analysis. Maternal/cord paired samples with sufficient serum volumes were utilized for the current study. Opportunistic sampling from the larger MACE cohort was performed. A total of 58 maternal- child pairs were included in this study. These samples were selected as serum blood samples were available for both maternal third trimester and cord blood, as well as the required demographic and clinical information. Inclusion and exclusion criteria were as per MACE study and are detailed briefly below. Inclusion criteria: pregnant females >18 years in third trimester of pregnancy attending one of the antenatal clinics as described earlier. Exclusion criteria: presence of any of the following clinical conditions pre-eclampsia, hypertension, placenta praevia, diabetes, genital tract infection and multiple pregnancies (32). Vitamin D measurement and supplementation in pregnancy is not part of routine practice guidelines in the South African public health care system.

### 2.2 Bisphenol A and Bisphenol glucuronide analysis

The methods used for determination of BPA and BPA-g levels has been previously described in detail (32). Briefly BPA and BPA-g levels were carried out using the AB Sciex 4500 triple quadrupole mass spectrometer equipped with an Agilent 1260 Ultra high-performance liquid chromatography (uHPLC) system. Analytes of interest were separated on a Phenomenex C18 column (2.1 x 50 mm, 1.6 um). A 3-minute linear gradient was used from 10-100% of acetonitrile in water followed by a hold for 1 minute at a flow rate of 0.4 ml/min. Serum samples were prepared using 50 µl of serum mixed with 100 µl acetonitrile containing the internal standards deuterated 5 ng/mL BPA (d6BPA, Cambridge Isotope Laboratories, Andover, MA) and 5 ng/ml ^13^C_12_ BPA-g (Sigma-Aldrich Gmbh, Munich, Germany. Electrospray ionization in negative modes was used for the measurement of each analyte. Qualifier and quantifier single reaction monitoring (SRM) transitions were used for both BPA and BPA-g. The limit of detection (LOD) and limit of quantification (LOQ) for BPA and BPA-g were calculated based on signal-to-noise (S/N) ratios of 3:1 and 10:1, respectively, The LOD and LOQ for both BPA and BPA were 0.12 ng/mL and 0.4 ng/mL respectively.

### 2.3 Determination of 25 hydroxy vitamin D levels

Following BPA and BPA-g analysis, the fifty-eight sample pairs were analyzed for Vitamin D. 25 OHD3 and 25 OHD2 were measured in maternal and cord serum by high performance liquid chromatography using a commercial kit, ClinRep (Recipe, Munich, Germany). Total25 OHD is the sum of the measured D3 and D2. The intra assay CV for 25OHD3 and D2 ranged from 0.9–4.9% and the inter-assay CV ranged from 3.0– 4.9%. The limit of detection was 2.5 nmol/l for 25(OH)D_3_ and 7.5 nmol/L for 25(OH)D_2_. Further details on this method have been previously published (34). Total Vitamin D levels of < 50 nmol/L were deficient and values between 50 and 75 nmol/l were classified as insufficient as defined by Endocrine Society practice guidelines (35). (Refer to **Table 1**).

**Table 1:** Endocrine Society Practice guidelines classification of vitamin D status in relation to 25(OH)D levels.

25(OH) Vitamin D Concentration (nmol/L)	Classification
<50 nmol/L	Deficient
>50 -<75 nmol/L	Insufficient
≥ 75 nmol/L	Sufficient

### 2.4 Statistical analysis

Univariate analyses were performed for maternal and newborn characteristics, including means and standard deviations or median and range for continuous variables. Data was assessed for normality using the Shapiro-Wilk test. Non-parametric tests Kruskal Wallis test, Spearman’s correlation or Wilcoxon signed rank test for performed for the univariate analysis. Kruskal-Wallis analysis (or one way ANOVA for parametric data) was performed to determine if any statistically significant difference could be identified between the two sexes for cord blood BPA, BPA-g and total 25OHD levels. A *p* value of <0.05 was considered significant. Analysis of covariance (ANCOVA) was used to assess for confounding variables (maternal BMI, infant mass and sex, gestational, seasonal variation), with the dependent continuous variable being total 25OHD levels and independent variable either maternal or cord BPA-g levels. Continuous variables with values below detectable limits were excluded from further data analysis or treated as categorical data. Statistical analysis was performed on Medcalc statistical software program version 18.11(Medcalc, Belgium).

### 2.5 Ethical approval

The research has complied with all the relevant national regulations, institutional policies and in accordance the tenets of the Helsinki Declaration and has been approved by the authors’ institutional review board or equivalent committee. Ethical clearance for this study was obtained from the Biomedical Research and Ethics Committee (BREC) of the University of KwaZulu-Natal (Ethics Clearance Certificate BE 597/16).

## 3 Results

A total of 58 maternal-cord pairs were analyzed for 25OHD. **Table 2** summarizes the demographic data, associated 25OHD levels and other baseline characteristics of the cohort. The 58 maternal participants, described in this study were all self-declared non- smokers. There was no statistically significant sex difference for any of the parameters presented.

**Table 2:** Baseline characteristics of cohort*.

Maternal (n=58)		Child (n=58)				
Characteristic		Characteristic	ALL	Male (n=36)	Female (n=22)	*p value*
Age (years)	25.0 (17-40)	Gestation (weeks)	38 (33-41)	38 (32-41)	38 (33-41)	p=0.87
BMI (kg/m^2^)	32.8 (9.4)	Birth weight (grams)	2695 (541)	2588 (502)	287 (569)	p=0.06
Gestation when samples takenc(weeks)	30 (3)	Length (cm)	49 (33-56)	49 (40-53)	49 (33-56)	p=0.78
		Head circumference (cm)	33.5 (27-47)	34 (27-44)	33 (30-47)	p=0.95
Total 25OHD levels (nmol/L)	55.7 (12.9)	Cord blood Total 25 OHD levels (nmol/L)	54.7 (19.1)	56.6 (19.8)	51.5 (18.0)	p=0.34
BPA (ng/mL)	0.8 (0.4-6.4)	Cord blood BPA (ng/mL)	0.91 (0.4-8.0)	0.7 (0.4-7.9)	1.3 (0.4-6.9)	p=0.60
BPA-g (ng/mL)	3.9 (0.15-21.8)	Cord blood BPA-g (ng/mL)	4.1 (0.34-26)	3.7 (0.34-26.0)	4.3 (0.65-21.3)	p=0.74

*Note data reported as mean (SD) values for normally distributed parameters and as median (range) values for non- normally distributed parameters. *P* values refer to the difference between male and female children.

### 3.1 BPA and BPA-g findings

BPA was detected in 17 of the 58 maternal samples (median 0.8 ng/mL; range 0.4-6.4) and 15 cord blood samples (median 0.91 ng/mL; range 0.4-8.0). The remainder of samples did not have detectable levels of BPA and these were not included in any continuous variable analyses. These findings are in keeping with the previously published data for the larger cohort where more than 70% of both maternal and cord samples had lower than detectable BPA concentrations (32). BPA-g was present at detectable concentrations in all 58 maternal-child pairs. This can be explained by the significantly shorter half-life of BPA in comparison to BPA-g. BPA-g in serum reflects the cumulative dose of BPA exposure over time (30).

### 3.2 Maternal and cord total 25OHD levels

Only four (2 pairs) of the 116 serum samples had detectable 25OHD2 levels. Thus, analysis performed examined total 25OHD levels. Maternal total 25OHD levels ranged from 29.5 to 94.4 nmol/L and cord levels from less than the LOD to100.6 nmol/L. Thirty four percent (n=20) of maternal samples and 50% (n=29) of cord blood samples had deficient (i.e < 50 nmol/L) and insufficient 25OHD levels. Twenty one percent (n=12) of maternal-child pairs were 25OHD deficient and 33% of the pairs were considered insufficient (>50 - 75 nmol/L). **Table 3** below provides further information regarding the categorization of 25OHD levels across the maternal-child pairs.

**Table 3:** Categorization of 25OHD levels (based on Endocrine Society Practice Guidelines) across the maternal-child pairs (n=58 pairs).

	Both maternal and cord blood samples deficient (<50 nmol/L)	Both maternal and cord blood samples insufficient (<75 nmol/L)	Only maternal sample deficient (<50 nmol/L)	Only cord sample deficient (< 50 nmol/L)	Both maternal and cord blood sufficient (≥ 75 nmol/L)
N (%)	12 (21%)	19 (33%)	8(14%)	17 (29%)	2 (3%)
Serum BPA-g levels per 25OHD categorization					
	Deficient	Insufficient		Sufficient	p value
Maternal BPA-g (ng/mL)	4.58 (1.03-11.7) (n=20)	3.29 (0.15-21.8) (n=29)		3.5 (3.23-6.35) (n=9)	p=0.075
Cord BPA-g (ng/mL)	4.14 (0.357-26) (n=29)	3.91 (0.34-9.85) (n=23)		2.62 (2.15-3.4) (n=6)	p=0.036

Note data reported as median (range).

### 3.3 Relationship between maternal, cord blood BPA/BPA-g and vitamin D levels

Spearman’s rank correlation showed a positive correlation of cord blood and maternal BPA-g levels (r=0.74 p <0.001) as well as with cord blood and maternal BPA levels (p=0.008). BPA levels were detected in a smaller percentage of the cohort as compared to BPA-g (maternal n=17; cord n=15). Cord blood 25OHD directly correlated with maternal 25OHD levels (Spearman’s correlation coefficient (r=0.5 p=0.002) 

**Figure 1:**
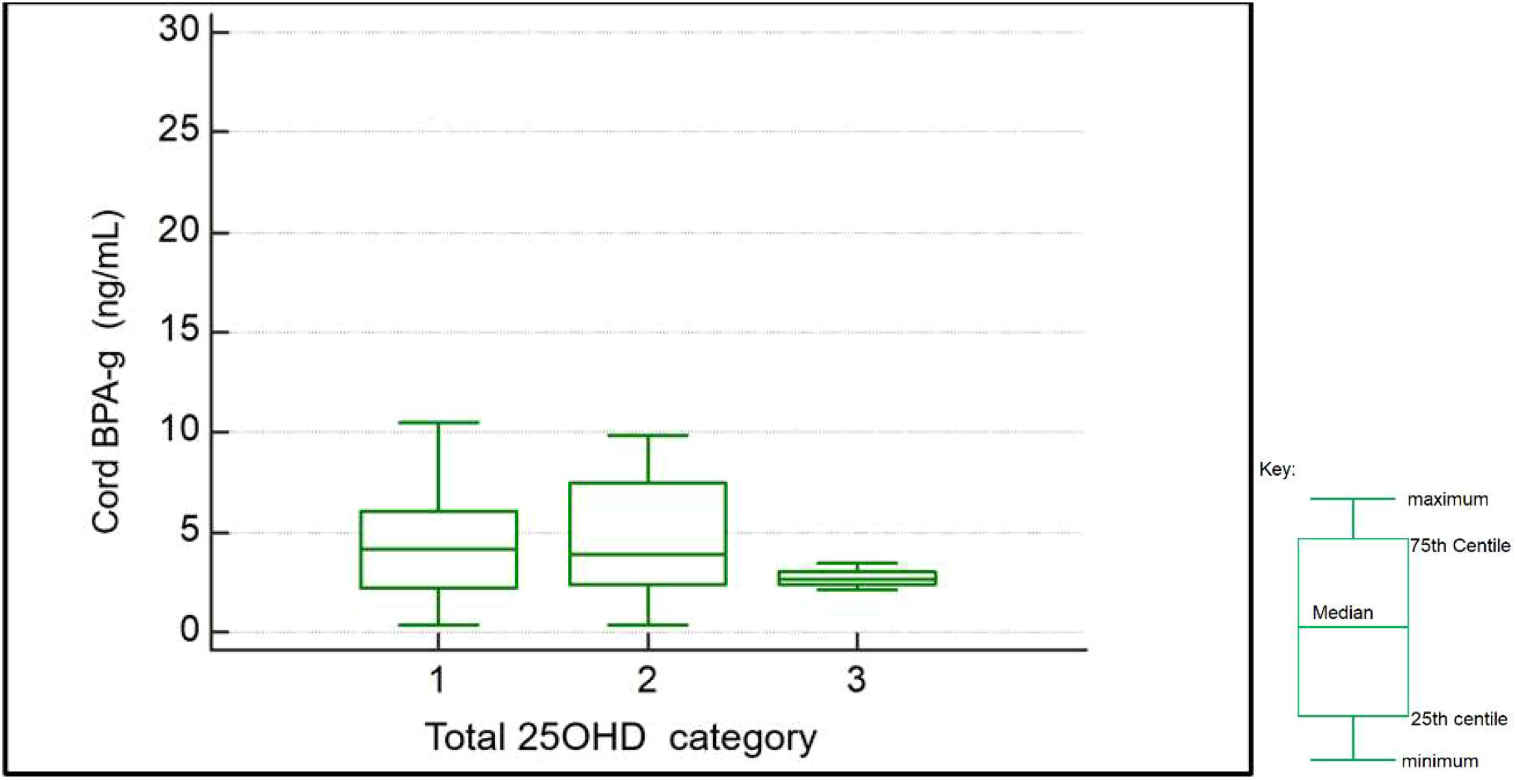
Box and whiskers plot- showing BPA-g levels per total 25OHD category as found in cord blood samples (1: deficient n=29; 2: insufficient n=23; 3: sufficient n=6).

On rank correlation maternal BPA (slope=-1; p=0.18) and cord BPA (slope =-1,2 p=0.4) showed a negative but not statistically significant relationship with cord total 25OHD levels. Due to the small number of samples with detectable BPA levels further analysis was not performed. Spearman rank correlation also showed a slight negative relationship between cord total 25OHD levels and maternal (slope -0.2 p=0.6) and cord BPA- g (slope -0.3 p=0.6) levels but these were not statistically significant. Kruskal Wallis analysis of maternal and cord BPA-g levels across total 25OHD categories was performed (refer **Figure 1**). This showed no statistical difference of BPA-g median levels (for maternal BPA-g p=0.75 and cord levels p=0.36) between study participants classed as having deficient, insufficient or sufficient vitamin D.

Notably a trend for higher BPA-g levels was observed in both maternal and cord bloods for those with deficient and insufficient 25OHD. (see **Table 3**) Analysis of covariance was performed to determine the effect of other confounders on this relationship. We wished to examine for any significant association with the presence of BPA or its metabolite on cord or maternal total 25OHD levels (continuous variable) when controlling for these confounders. Of note cord (p=0.033) and maternal BPA-g (p=0.04) levels showed statistically significant associations with cord total 25OHD levels. Maternal BMI, gestational age of infant, and infant sex did not show any statistically significant relationship with cord total 25OHD levels on ANCOVA analysis. On ANCOVA analysis maternal total 25OHD levels showed no significant relationship with BMI (p=0.9), age (p=0.7), weeks of gestation when sample taken (p=0.9) or maternal BPA-g levels (p=0.8)

### 3.4 Infant anthropometric parameters and vitamin D levels

On Spearman’s rank correlation neither head circumference nor length were significantly correlated with either maternal (p=0.24 and p=0.40 respectively) or cord blood (p=0.7 and p=0.2 respectively) total 25OHD levels. Whilst on ANCOVA analysis birth weight showed a statistically significant association with cord total 25OHD levels (p=0.05), Spearman’s rank correlation was not significant (p=0.15).

### 3.5 Influence of seasonal variation

Based on the month samples were taken, data was classified with regards to season (winter, summer, spring and autumn). There were 24 maternal-cord pairs taken in winter, 20 pairs in summer, 9 pairs in spring and 5 pair taken in autumn. Mean values for samples taken in autumn were trending higher for both cord and maternal total 25OHD versus the other seasons, with levels 15-30% higher. Seasonal variation lacked statistical significance on one way ANOVA analysis with regards to association with cord blood vitamin D levels (p=0.07) and maternal vitamin D levels (p=0.06). The lack of significance may be attributed to the small sample numbers for each seasonal category.

## 4 Discussion

The most striking finding in this pilot study was the association (after correction for confounders) between a known endocrine disruptor (BPA metabolite BPA-g) and cord total 25OHD levels. Two previous studies have described a significant negative relationship with urine BPA metabolites and 25OHD in women, pregnant women and cord blood (29, 36). These studies did not examine serum BPA or BPA-g levels in their cohorts. Cord BPA-g reflects the cumulative dose of exposure of the fetus to maternal BPA in late pregnancy (30) and as such is more likely a better surrogate of *in-utero* exposure than maternal urinary BPA. However, no studies to date have reported on the relationship of serum BPA metabolites including BPA-g on total 25OHD levels. Furthermore, the relationship between maternal and cord levels supports the evidence for antenatal BPA exposure being transferred to the newborn (32), while maternal 25OHD levels influence that of the growing infant (11–14).

The significant role 25OHD plays in maternal, fetal and childhood health and beyond, has been previously described (2–10). An inverse relationship between vitamin D and serum BPA has been reported, across both adult males and females, in a single study (37). However, BPA-g levels were also not measured in this instance. BPA-g, which is the major metabolite of BPA was widely thought of inert as it is unable to bind to steroid receptors however recent evidence in animal studies suggest that it may deconjugate to expose the fetus to BPA even though adequate conjugation of BPA has occurred after maternal intake (31). Animal studies have shown that whilst BPA-g levels in maternal serum may be highly variable, levels in cord serum remain steady reflect the cumulative dose of BPA received by the fetus during late pregnancy and thus acts as a surrogate of exposure (30). This may partly explain the presence of a significant association with cord total 25 OHD levels but an absence of this finding with maternal 25OHD.

The presence of BPA in the environment is ubiquitous. There has been much research on how the intrauterine environment and exposure of individuals preconception and pregnant females during the gestational period affect eventual infant outcomes and disease occurrence throughout life (25). This pilot study demonstrated lower median levels of maternal BPA (0.80 ng/mL) which have also been reported in previous studies performed in industrialized nations. Padmanabhan et al. reported values of serum maternal BPA ranging from undetectable (<0.5 ng/mL) to 22.3 ng/mL with a mean value of 5.9 ng/mL in a US cohort (38). A larger study of 300 participants in South Korea reported BPA concentrations from non-detectable to 66.48 ng/mL in pregnant women and from non-detectable to 8.86 ng/mL in umbilical cord blood (39). In this current pilot study the majority of maternal serum (71%) and cord blood samples (74%) had BPA levels lower than the detectable limit of the assay. Detection rates in other studies ranged from 14 to 17% above lower limit of detection for serum maternal BPA (40, 41). One study reported a mean maternal serum BPA-g of 0.36 ng/mL and mean cord BPA-g of 0.09 ng/mL, whilst another reported third trimester maternal BPA-g values of 6.77 ng/mL (42, 43). Both studies used study participants from developed nations. Differences in maternal diet as well as environmental exposure may play a role in the variability of findings with regards to BPA and BPA-g concentrations in different populations.

There is sparse data arising from the limited mechanistic studies examining the effect of BPA on the vitamin D endocrine system. Animal studies have demonstrated that BPA can disturb calcium metabolism by influencing the expression of vitamin D – dependent calcium binding protein (44–46). The study by Otsuka et al. also showed an inverse relationship with serum calcium levels and BPA in pregnant mice (45). Another possible mechanism of BPA on Vitamin D metabolism is via an effect on metabolizing enzymes; either by changing the expression of cytochrome P450 enzymes responsible for steroid metabolism or affecting messenger RNA (mRNA) expression (46, 47). A recent study examining the effect of BPA exposure on male and female rats beginning from post-natal day 9, for 91 days demonstrated that BPA increased urinary excretion of 25OHD3 thus decreasing vitamin D levels in serum (48). This is further suggestive of mechanistic effect of BPA on vitamin D metabolism.

The findings of this study demonstrated that a significant proportion of pregnant women and neonates have suboptimal 25OHD levels. More than 50% of the maternal and cord blood samples were either deficient or had insufficient levels of total 25OHD. Whilst there was a direct and significant correlation between maternal and cord blood total 25 OHD levels, the correlation was moderate (R=0.5 p=0.002) in this study as compared to some previous studies. Jacquemyn et al. reported a correlation of R=0.91 in a multi-ethnic cohort from Belgium (49). One possibility to be considered to explain the moderate correlation in this study is the effect of BPA exposure on the cohort.

This pilot study utilized the Endocrine Society practice guidelines for determination of cut-offs for interpretation of Vitamin D levels in the maternal cord pairs (35). There is currently no consensus with regards optimal cut-offs in pregnant women or infants and various cut -offs recommended by the various authorities and societies. The Institute of Medicine (IOM) guidelines stipulate values ≥ 50 nmol/L as being sufficient (2). Using this cut off: 31% (n=18/58) of mothers and 41% of new-borns (n=24/58) would be vitamin D insufficient. However, these cut points are based solely on sufficiency for adequate bone health and not for the other aspects of health and physiological functions that have been linked to vitamin D status (50). These functions include neurodevelopment, immune, cardio-metabolic, reproductive function and protection against cancer (50–53).

BPA exposure has itself been linked to negative sequelae affecting the same physiological systems as well as in the development of malignancy (25, 54). These guidelines have also been largely developed in European/North American populations and have not been verified in other populations with randomized controlled trials (50). Seasonal variation was also noted with significantly higher mean values noted in autumn. This is in keeping with previous reports in southern hemisphere cohorts (34, 55).

One of the strengths of this study is the use of the specific and sensitive HPLC methodology to measure the 25 hydroxy vitamin D2 and D3 levels. Many of the previous studies examining the relationship between maternal and cord Vitamin D utilized immunoassay-based techniques. Immunoassay is more susceptible to interferences from compounds of similar structure and from heterophile antibodies (56). Race has been reported as a significant confounder on Vitamin D levels (57). This pilot study cohort was homogenous with regards to race as all participants were Black African.

There are limitations to the current study. This includes the cross-sectional nature of the study and the small sample size. As BPA and BPA-g levels were not followed over time during the course of the pregnancy we cannot be certain to what degree the measured maternal serum and cord blood BPA/BPA-g levels reflect the actual exposure throughout pregnancy. A further limitation is that the majority of sample- pairs did not have detectable serum BPA levels. However, all sample pairs had detectable BPA-g levels which has been demonstrated to be a more reliable surrogate marker of BPA exposure. The current study did not take into consideration the possible confounding effect of dietary food intake and use of sunscreen on the maternal vitamin D levels. However, in this population cohort due to low socio-economic status use of both sunscreen and vitamin D supplementation is unlikely. Additionally, the almost absence of detectable 25OHD2 levels amongst this study population is an indication that vitamin D supplementation did not occur during pregnancy.

This is the first study to the authors’ knowledge to examine the relationship between serum BPA as well as BPA-g with 25 hydroxy vitamin D levels in maternal-child pairs. Our findings suggest a relationship between BPA exposure and Vitamin D levels in the intra-uterine period. Follow up is requited to understand causality or associations with development of disease in the BPA exposed infants. Further studies are required to examine the mechanistic relationship of exposure to endocrine disruptors like BPA and its effect on Vitamin D – to better evaluate and understand the health consequences in humans.

## Data Availability

The raw data supporting the conclusions of this article will be made available by the authors, without undue reservation.
